# Large-Scale Analysis of Zipf’s Law in English Texts

**DOI:** 10.1371/journal.pone.0147073

**Published:** 2016-01-22

**Authors:** Isabel Moreno-Sánchez, Francesc Font-Clos, Álvaro Corral

**Affiliations:** 1 Centre de Recerca Matemàtica, Edifici C, Campus Bellaterra, E-08193 Barcelona, Spain; 2 Departament de Matemàtiques, Facultat de Ciències, Universitat Autònoma de Barcelona, E-08193 Barcelona, Spain; National Scientific and Technical Research Council (CONICET)., ARGENTINA

## Abstract

Despite being a paradigm of quantitative linguistics, Zipf’s law for words suffers from three main problems: its formulation is ambiguous, its validity has not been tested rigorously from a statistical point of view, and it has not been confronted to a representatively large number of texts. So, we can summarize the current support of Zipf’s law in texts as anecdotic. We try to solve these issues by studying three different versions of Zipf’s law and fitting them to all available English texts in the Project Gutenberg database (consisting of more than 30 000 texts). To do so we use state-of-the art tools in fitting and goodness-of-fit tests, carefully tailored to the peculiarities of text statistics. Remarkably, one of the three versions of Zipf’s law, consisting of a pure power-law form in the complementary cumulative distribution function of word frequencies, is able to fit more than 40% of the texts in the database (at the 0.05 significance level), for the whole domain of frequencies (from 1 to the maximum value), and with only one free parameter (the exponent).

## Introduction

Zipf’s law constitutes a striking quantitative regularity in the usage of language [1–4]. It states that, for a large enough piece of text, the frequency of use *n* of any word decreases with its rareness *r* in the text in an approximately hyperbolic way, i.e., *n* ∝ 1/*r*, where the symbol “∝” denotes proportionality. Technically, *r* is called the rank, and the most common (i.e., less rare) word is assigned *r* = 1, the second most common, *r* = 2, and so on. A slightly more general formulation includes a parameter in the form of an exponent *α*; then, the rank-frequency relation takes the form of a power law,

$$n\;\propto \frac{1}{r^{\alpha }}.$$ (1)

with the value of *α* close to one.

This pattern Eq (1) has been found across different languages, literary styles, time periods, and levels of morphological abstraction [2, 5–7]. More fascinatingly, the same law has been claimed in other codes of communication, as in music [8] or for the timbres of sounds [9], and also in disparate discrete systems where individual units or agents gather into different classes [10], for example, employees into firms [11], believers into religions [12], insects into plants [13], units of mass into animals present in ecosystems [14], visitors or links into web pages [15], telephone calls to users [16], or abundance of proteins (in a single cell) [17]. The attempts to find an explanation have been diverse [3, 16, 18–25], but no solution has raised consensus [21, 26–28].

Despite its quantitative character, Zipf’s law has been usually checked for in a qualitative way, plotting the logarithm of the frequency *n* versus the logarithm of the rank *r* and looking for some domain with a roughly linear behavior, with slope more or less close to −1. A more refined approach consists in fitting a straight line to the double-logarithmic plot by linear regression [29]. But several authors have recently pointed out the limitations of this method when applied to probability distributions [12, 30–32], and the advantages of using an asymptotically unbiased and minimum-variance procedure such as maximum likelihood (ML) estimation [32], whose solutions, moreover, are invariant under reparameterizations [33, 34]. One should consider then ML estimation as the most reliable procedure of estimation for parametric models (when a maximum of the likelihood does exist and the number of data is large).

Furthermore, for the particular case of linguistics, the search for Zipf’s law has been traditionally performed in very limited sets of texts (less than a dozen in a typical research article [6, 35], although in hundreds of languages [7]). More recently, however, large corpora have been considered –these are representative collections of different texts aggregated together into a single bag, so, instead of many separated texts one deals with one enormous mixed text. When “rare” words are not considered (rare in terms of the frequency, i.e., frequencies below some particular value), it seems that Zipf’s law still holds in these large collections, in the sense that Eq (1) is valid only for high frequencies [2, 36–39].

At present, there is agreement that Zipf’s law is a rough approximation in lexical statistics, but its range of validity is totally unknown, i.e., we ignore how good Zipf’s law is in order to account for the appearance of words, and for which texts it should work –and with which level of precision– and for which texts it should fail. We know, however, that this peculiar pattern seems to hold in hundreds of different languages, in concrete in those languages in which some electronic texts are available [7].

An extra difficulty emerges when one recognizes the ill-defined nature of Zipf’s law. In fact, the law has two formulations, with the first one being Eq (1), which just counts the frequency of words. For the sake of clarity, the words that are counted are referred to as word types, in order to distinguish them from each repetition, which is called a token. The second formulation of Zipf’s law arises when, after counting the frequency of word types, one performs a second statistics and counts how many values of the frequency are repeated, that is, how many word types have the same frequency. This means that the frequency *n* is considered the random variable. One can realize that the rank, when normalized by its maximum value in text, is just the empirical estimation of the complementary cumulative distribution function of *n*, and then, the derivative of the expression for *r*(*n*) (the inverse of Eq (1)) yields a continuous approximation for the probability mass function *f*(*n*) of the frequency *n*. From here one obtains another power law,

$$f\left(n\right)\;\propto \frac{1}{n^{\beta }},$$ (2)

with the new exponent *β* fulfilling *β* = 1 + 1/*α*, which yields values of *β* close to 2. The expression given by Eq (2) was in fact the first approach followed by Zipf’s himself [3], and is usually considered as equivalent to Eq (1) [3, 10, 15, 16, 35]; however, as it is derived in the continuum limit, both expressions can only be equivalent asymptotically, for large *n* [40]. Consequently, if one wants to be precise, a natural question follows: which one is the “true” Zipf’s law (if any)?

We cannot know a priori which of the two Zipf’s laws better describes real texts, but we can argue which of the two representations (that of *n*(*r*), Eq (1), or that of *f*(*n*), Eq (2)) is better for statistical purposes, independently of the functional dependency they provide. It is clear that the rank-frequency representation, given by *n*(*r*), presents several difficulties, due to the peculiar nature of the rank variable. First, in Ref. [41], Zipf-like texts were randomly generated following Eq (1), keeping the original ranks “hidden” (as it happens in the real situation), and it was found that the rank reconstructed from the sample deviated considerably from the original ranks when these were taking large values (which for a power law happens with a high probability). The resulting ML estimations of the exponent *α* were highly biased and the Kolmogorov-Smirnov test rejected the power-law hypothesis, although the original ranks were power-law indeed.

One might argue that the problem could be escaped by using an upper truncated power-law distribution (introducing then an additional parameter for the truncation), in order to avoid the inconsistency of the rank representation for high values. But a second problem is that the rank is not a true random variable [42], as its values are assigned a posteriori, once the sample (i.e., the text) is analyzed. This means that the rank does not show “enough” statistical fluctuations, that is, if *r*_*a*_ < *r*_*b*_, then the frequency of *a* is always larger, by construction, than the frequency of *b*. This does not necessarily happen for a true random variable. The negative correlation between the variable and its frequency of occurrence makes the power-law hypothesis harder to reject. In fact, inflated *p*-values (not uniformly distributed between 0 and 1) have been found when fitting truncated power laws to simulated power-law rank-frequency representations [41]. This problem could still be avoided by choosing a low enough upper truncation parameter (yielding a very short range of ranks, for which the fluctuations would be very little) but at the expense of disregarding an important part of the data.

A third inconvenience is the impossibility, due to normalization, that a non-truncated power law comprises values of the *α*—exponent smaller than 1. This yields the necessity of introducing a truncation parameter that may be artifactual, i.e., not present in the real system. All this leads to the conclusion that the most reliable method of parameter estimation (ML, in a frequentist framework) cannot be directly applied to the rank-frequency representation. In contrast, the representation in terms of the distribution of frequencies is devoid of these problems [41], as *n* is a well-defined random variable, and this will be the representation used in this paper for statistical inference. Nevertheless, for alternative arguments, see Ref. [43].

The purpose of this paper is to quantify, at a large, big-data scale, different versions of Zipf’s law and their ranges of validity. In the next section, we present and justify the three Zipf-like distributions we are going to fit, and we briefly explain the selected fitting method and the goodness-of-fit test. The corpus of texts under consideration is also detailed. The subsequent section presents the results, with special attention to their statistical significance and their dependence with text length. Finally, we end with a discussion, conclusions and some technical appendices.

## Zipf-like Distributions

As implicit in the introduction, and in contrast with continuous random variables, in the discrete case a power law in the probability mass function *f*(*n*) does not lead to a power law in the complementary cumulative distribution or survival function *S*(*n*), and vice-versa. Let us specify our definition for both functions, *f*(*n*) = Prob[frequency = *n*] (as usual), and *S*(*n*) = Prob[frequency ≥ *n*] (changing, for convenience, the usual strict inequality sign by the non-strict inequality). Then, the relation between both is *f*(*n*) = *S*(*n*) − *S*(*n* + 1) and $S\left(n\right)=\sum_{n^{\prime }=n}^{\infty }f\left(n^{\prime }\right)$.

We consider that the values the random variable takes, given by *n*, are discrete, starting at the integer value *a*, taking values then *n* = *a*, *a* + 1, … up to infinity. In this study we will fix the parameter *a* to *a* = 1, in order to fit the whole distribution and not just the tail. Then, although for large *n* and smooth *S*(*n*) we may approximate *f*(*n*) ≃ −*dS*(*n*)/*dn*, this simplification is clearly wrong for small *n*. Note that the simplification leads to the implication that a power law in *f*(*n*) leads to a power law in *S*(*n*), and vice-versa, but this is clearly wrong for small values of *n* in discrete distributions. The simplification also lies in the equivalence between Eqs (1) and (2), assuming that *S*(*n*) is proportional to the rank and inverting Eq (1).

For the first distribution that we consider, the power-law form is in *f*(*n*), then,

$$f_{1}\left(n\right)\;=\frac{1}{\zeta \left(\beta ,a\right)n^{\beta }}.$$ (3)

This is just the normalized version of Eq (2), and then,

$$S_{1}\left(n\right)\;=\frac{\zeta (\beta ,n)}{\zeta (\beta ,a)}$$

with *β* > 1 and $\zeta \left(\beta ,a\right)=\sum_{k=0}^{\infty }{\left(a+k\right)}^{-\beta }$ the Hurwitz zeta function, which ensures normalization of both expressions of the distribution (*f*_1_(*n*) and *S*_1_(*n*)). A preliminary analysis of texts in terms of this distribution was done in Ref. [44].

In contrast, when the power law is in *S*(*n*), this leads to our second case,

$$f_{2}\left(n\right)\;={\left(\frac{a}{n}\right)}^{\beta -1}-{\left(\frac{a}{n+1}\right)}^{\beta -1}$$ (4)

and

$$S_{2}\left(n\right)\;={\left(\frac{a}{n}\right)}^{\beta -1}$$

with *β* > 1 again. Note that this corresponds to a power law in the empirical rank-frequency relation. As *S*_2_(*a*) = 1 this ensures normalization of *S*_2_(*n*), and also of *f*_2_(*n*), which is derived from *S*_2_(*n*).

Finally, it is interesting to consider also the frequency distribution derived by Mandelbrot [40] when ranks are generated independently from a power law in Eq (1), which is,

$$f_{3}\left(n\right)\;=\frac{\left(\beta -1)\Gamma (a\right)}{\Gamma (a+1-\beta )}\frac{\Gamma (n+1-\beta )}{\Gamma (n+1)}$$ (5)

and

$$S_{3}\left(n\right)\;=\frac{\Gamma (a)\Gamma (n+1-\beta )}{\Gamma (n)\Gamma (a+1-\beta )},$$

with 1 < *β* < 2, and $\Gamma \left(\gamma \right)\;=\int_{0}^{\infty }x^{\gamma -1}e^{-x}dx$ denotes the gamma function [45]. In this case the power law is the underlying theoretical rank-frequency relation *n*(*r*). Note that *f*_3_(*n*) can be written as

$$f_{3}\left(n\right)=\frac{B(n+1-\beta ,\beta )}{B(a+1-\beta ,\beta -1)}$$

using the beta function [45], *B*(*x*, *y*) = Γ(*x*)Γ(*y*)/Γ(*x* + *y*), with an analogous expression for *S*_3_(*n*) (nevertheless, do not confuse this distribution with the beta distribution).

In all three cases it is easy to show that we have well-defined, normalized probability distributions, when *n* takes values *n* = *a*, *a* + 1, …, with *a* being a positive integer. Moreover, in the limit *n* → ∞ all of them yield a power-law tail, *f*(*n*) ∝ 1/*n*^*β*^, so *β* will be referred to as the power-law exponent. Indeed, it is easy to show that

$$f_{2}\left(n\right)\overset{n\to \infty }{\to }\left(\beta -1\right)\frac{a^{\beta -1}}{n^{\beta }},$$

whereas

$$f_{3}\left(n\right)\overset{n\to \infty }{\to }\frac{\left(\beta -1)\Gamma (a\right)}{\Gamma \left(a+1-\beta \right)n^{\beta }}$$

using Stirling’s formula [45]. The main difference between the three distributions is in the smaller values of *n*, taking *f*_2_(*n*) a convex shape in log-log-scale (as seen “from above”); *f*_3_(*n*) a concave one; and *f*_1_(*n*) being somehow in between, as it is neither concave nor convex.

## Methodology and Data

In order to fit these three distributions to the different texts, and test the goodness of such fits, we use maximum likelihood estimation [46] followed by the Kolmogorov-Smirnov (KS) test [47]. The procedure seems similar to the one proposed in Ref. [12], but as *a* is fixed here, the problems resulting from the search of the optimum *a*[34, 48] do not arise in this case.

The method of ML estimation proceeds in the following simple way. Given a set of data {*n*_*i*_} with *i* = 1, 2, …*N*, and a probability mass function parameterized by *β*, denoted as *f*(*n*; *β*) = *f*(*n*), the log-likelihood function is obtained as

$$l\left(\beta \right)\;=\sum_{i=1}^{N}\ln f\left(n_{i};\beta \right).$$ (6)

We are assuming that the data points *n*_*i*_ are independent from each other, in other words, we are calculating the likelihood that the data are generated independently from *f*(*n*; *β*). The ML estimation of *β* is obtained as the value of *β* which maximizes *l*(*β*); we undertake this numerically, using Brent’s method in the range 1 < *β* ≤ 4 [47]. In the case of the distribution *f*_1_ the log-likelihood function takes the simple form *l*_1_(*β*)/*N* = −ln(*ζ*(*β*, *a*)) − *β* ln*G*, with *G* the geometric mean of the set {*n*_*i*_}. For the other distributions no closed-form expression is possible and we use Eq (6) directly.

As mentioned, the goodness-of-fit test is done through the Kolmogorov-Smirnov statistic [12, 47], in the discrete case [49], for which the *p*-value is calculated from Monte Carlo simulations (due to the fact that, as the value of the exponent is calculated from the same data is going to be tested, the procedure would be biased towards the empirical value and the theoretically computed *p*-value [47] would be inflated). In this paper we use 100 Monte Carlo simulations for each test. The proper simulation of the 3 distributions is explained in the Appendix. Remember that a small enough *p*-value leads to the rejection of the fit. Although we perform multiple testing, we do not incorporate any Bonferroni-like correction [50–52], due to the fact that these corrections increase the number of non-rejected null hypotheses (that is, decrease the number of type I errors), inflating the performance of the fits, in the case of goodness-of-fit tests. Without Bonferroni-like corrections, our acceptance (i.e., non-rejection) of the fits is more strict.

In order to check the consistency of our fitting procedure, we also perform a direct comparison of models through the likelihood ratio (LR) test [12, 53]. Taking distributions *f*_1_ and *f*_2_, the log-likelihood-ratio between both is

$$R_{1,2}\;=\sum_{i=1}^{N}\left(\ln f_{1}\left(n_{i}\right)-\ln f_{2}\left(n_{i}\right)\right),$$

and, under the null hypothesis that both models are equally good to describe the data, *R*_1,2_ should be normally distributed with zero mean and a variance that can be estimated as *Nσ*^2^, with *σ*^2^ the variance of the random variable ln*f*_1_(*n*) − ln*f*_2_(*n*). Large absolute values of *R*_1,2_ will lead to the rejection of the null hypothesis.

In order to apply this methodology we consider a set of 36 813 texts in UTF-8 encoding downloaded from the Project Gutenberg database (accessed July 2014 [54]). These texts correspond to different languages, styles, and time periods, although most of them are works of literature from the Western cultural tradition [55]. First of all, parts of text that do not pertain to the piece under consideration (copyright notes, headers,…) are removed by an automatized process. Books that have not been filtered in this step (mainly because they do not have standard delimiters) are discarded. After this, we still keep 98.2% of the total (i.e., 36 147). To perform our study, we restrict ourselves to the subset of texts in English, which represent the 86% of these 36 147 (i.e., 31 102).

**Fig 1:**
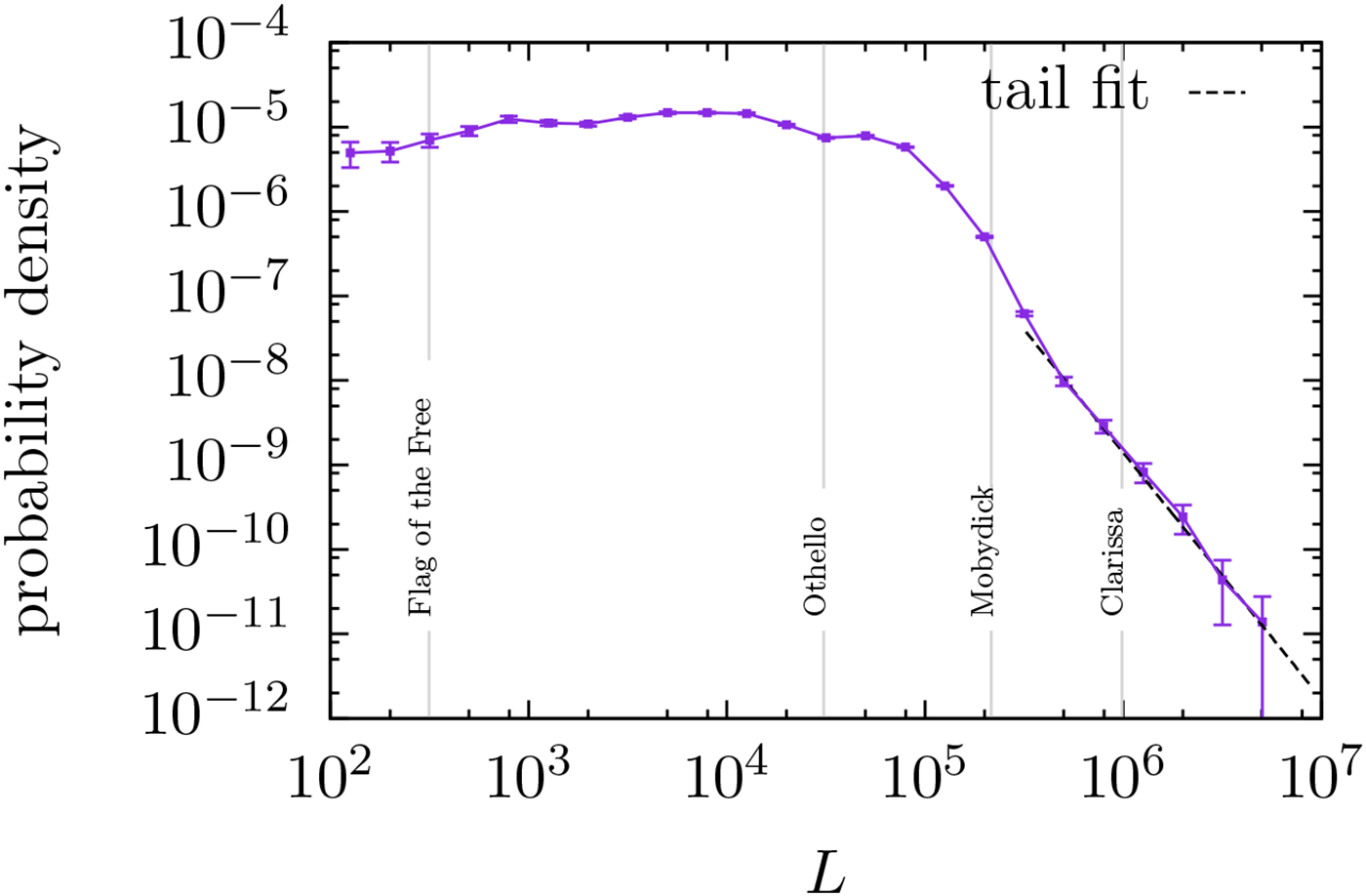
Estimation of the probability density function of text length *L* in the English Project Gutenberg database, using logarithmic binning (5 bins per decade). Texts with less than 100 tokens are not considered. A power-law fit of the tail [34] yields an exponent 2.92 ± 0.15.

An important characteristic of each text is its length, *L*, counting the number of word tokens contained in the text. It turns out to be that in the database *L* expands from very small values up to 4 659 068 tokens, with a distribution that is shown in Fig 1. Observe the roughly uniform distribution up to about *L* = 10^5^, and the decay afterwards.

For our analysis we consider only the 31 075 English texts that consist of more than 100 word tokens, as smaller texts would not have statistical value. For each of these texts we select then actual word types (punctuation signs, numbers and any character different from letters are not considered) to count their frequencies *n*, which will be our primary object of study. The values of these frequencies, for each text, are available on http://dx.doi.org/10.6084/m9.figshare.1515919, in order to facilitate the reproducibility of our results.

In summary, we apply the above described fitting and goodness-of-fit procedure –using ML estimation and the Kolmogorov-Smirnov test– to a total of 31 075 texts from the English Project Gutenberg database, using three different possibilities for the distribution of frequencies: *f*_1_ (Eq (3)), *f*_2_ (Eq (4)), and *f*_3_ (Eq (5)). This yields a total of 3 × 31075 fits and associated *p*-values, which we analyze and interpret in what follows.

## Results

**Fig 2:**
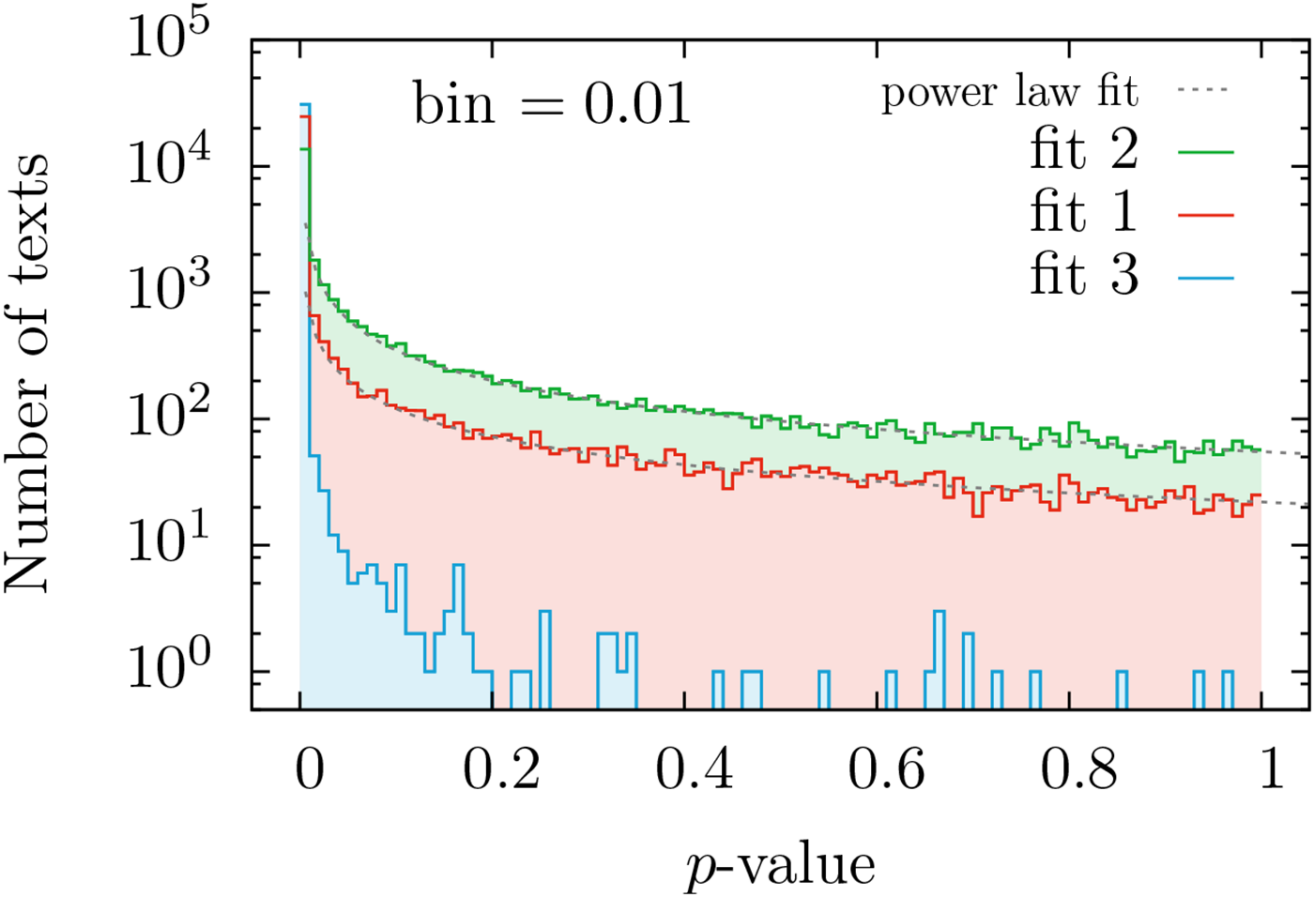
Histograms of *p*-values obtained when the Zipf-like distributions *f*_1_, *f*_2_, and *f*_3_ are fitted to the texts of the English Project Gutenberg. The histograms just count the number of texts in each bin of width 0.01. Note the poor performance of distribution 3 and the best performance of 2. Power-law approximations to the histograms for *f*_1_ and *f*_2_, with respective exponents 0.74 and 0.78, are shown as a guide to the eye.

**Fig 3:**
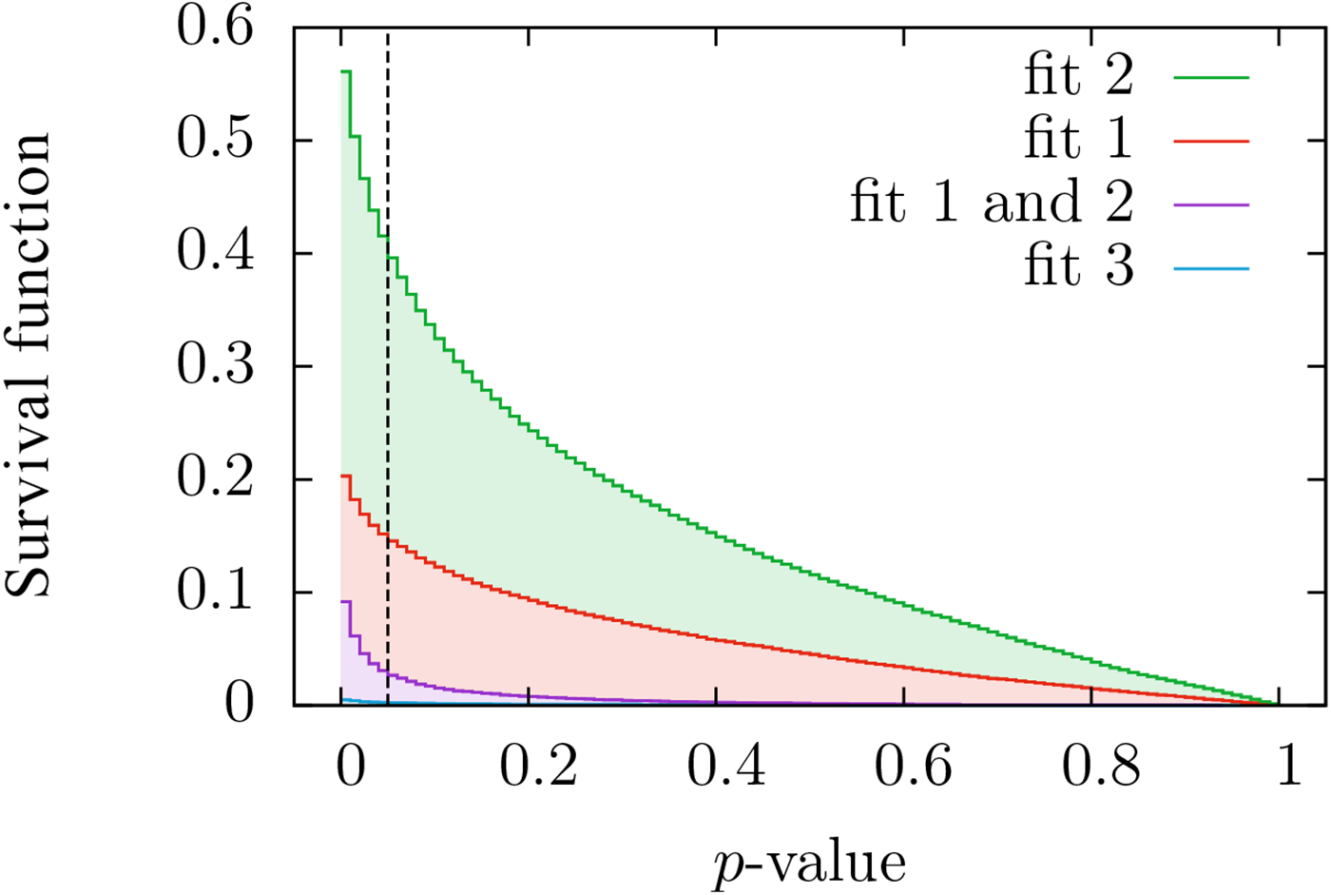
Complementary cumulative distributions (i.e., survival functions) of *p*-values obtained when our three distributions are fitted to the texts of the English Project Gutenberg. This corresponds, except for normalization, to the integral of the previous figure, but we have included a fourth curve for the fraction of texts whose *p*-values for fits 1 and 2 are both higher than the value marked in the abscissa. Note that the values of *p* can play the role of the significance level. The value for *p* = 0 is not shown, in order to have higher resolution.

Contrary to previous studies where the number of texts considered was, at most, in the order of tens, the large-scale approach taken in this work requires a statistical analysis of the fitting results, as a case-by-case interpretation is out of hand. We first focus on the distribution of *p*-values, see Figs 2 and 3. If *all* texts were truly generated by a mechanism following a given distribution, the corresponding *p*-values for that distribution would be uniformly distributed between zero and one [56] (see p. 28), [57] (see p.441). As seen in Fig 2, this is not the case and, furthermore, most texts have rather small *p*-values for the three fits; nevertheless, for distributions *f*_1_ and *f*_2_ there are still many texts that yield high enough *p*-values.

This implies that, although we cannot conclude that the whole database is generated by any of these distributions, these cannot be rejected as good descriptions for large subsets of the database. Regarding distribution *f*_3_, it is clear from the histogram of *p*-values that it can be discarded as a good description of the distribution of frequencies in any non-negligible subset of texts. So, from now on, we will concentrate on the remaining options, *f*_1_ and *f*_2_, to eventually quantify which of these better describes our corpus. In essence, what we are interested in is which version of Zipf’s law, either distribution *f*_1_ or *f*_2_, fits better a reasonable number of texts, and which range of validity these simple one-parameter distributions have.

**Fig 4:**
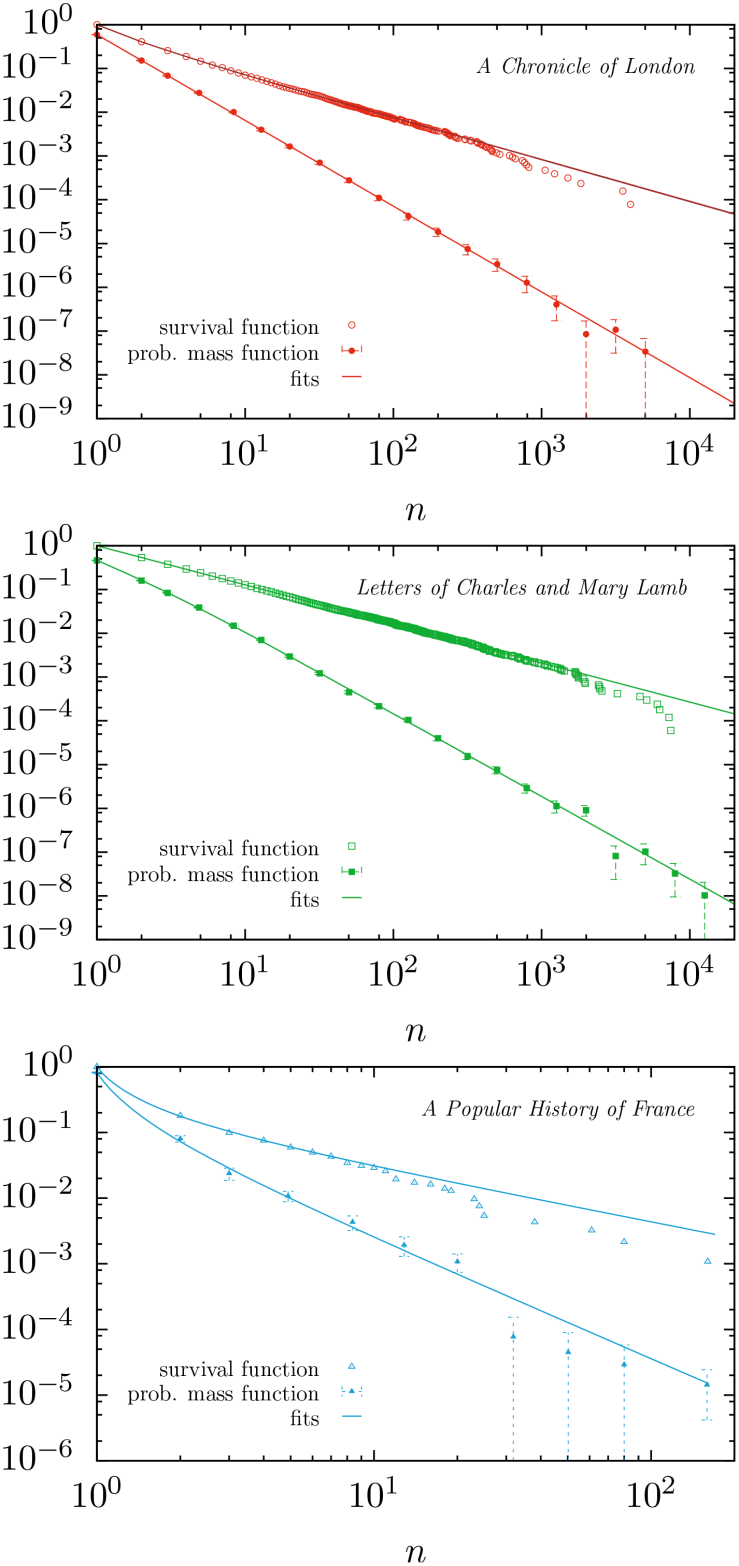
Complementary cumulative distribution and probability mass function of text frequencies, for: (a) *A Chronicle of London, from 1089 to 1483* (anonymous); (b) *The Works of Charles and Mary Lamb*, Vol. V, edited by E. V. Lucas; (c) *A Popular History of France from the Earliest Times*, Vol. I, by F. Guizot. These texts are the ones with the largest length *L* (83 720, 239 018 and 2 081 respectively) of those that fulfill *p* > 1/2, for fits 1, 2 and 3 respectively. The exponent *β* takes values 1.96, 1.89, and 1.82, in each case.

The outcome is that, independently of the significance level (as long as this is not below our resolution of 0.01 given by the number of Monte Carlo simulations), the ratio between the number of texts fitted by distribution *f*_2_ and those fitted by *f*_1_ is nearly constant, taking a value around 2.6. For example, considering significance level (i.e., minimum *p*-value) equal to 0.05, Fig 3 shows that distribution *f*_2_ fits about 40% of all texts, whereas distribution *f*_1_ fits just 15%. Both percentages include a 2.7% of texts that are fitted by both distributions simultaneously, although this number does not keep a constant ratio with the other two, rather, it decreases when the significance level is increased (as it is implicit in the values of Fig 3). Given that the aforementioned ratio of 2.6 is independent of the significance level, it is fair to say that distribution *f*_2_ provides, compared to *f*_1_, a better description of our database. As a visual illustration of the performance of the fits we display in Fig 4 the word frequency distribution of the longest texts that have *p* > 1/2, for distributions *f*_1_, *f*_2_ and *f*_3_.

**Fig 5:**
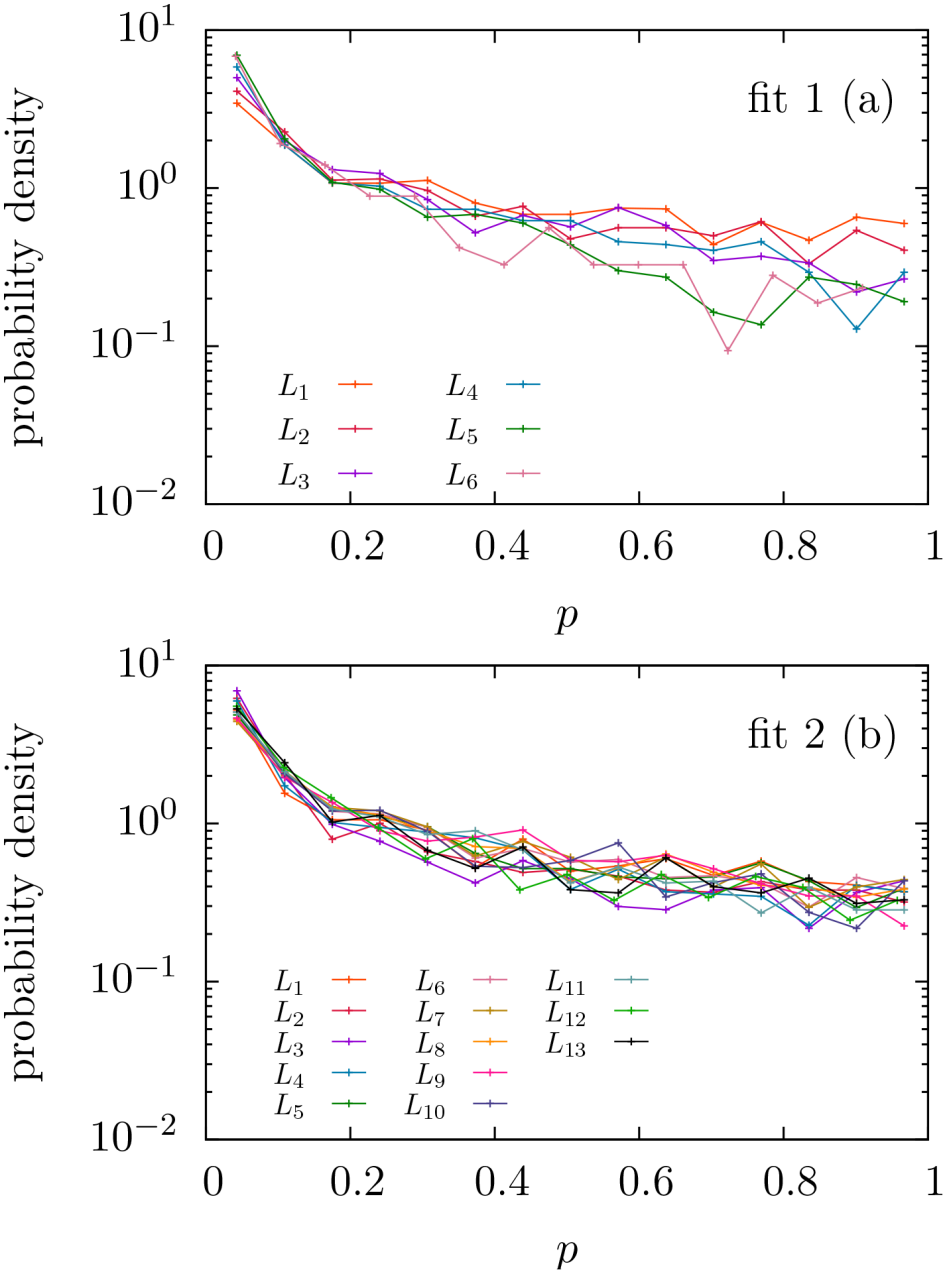
Estimated probability density functions of *p*-values conditioned to *p* ≥ 0.01 separating for different ranges of text length *L*. *p*-values correspond to the fitting of word frequencies to (a) distribution *f*_1_ and (b) distribution *f*_2_. We divide the distribution of text length into 15 intervals of 2 000 texts each. For distribution *f*_1_ only the first seven groups (up to length 34 400) are displayed (beyond this value we do not have enough statistics to see the distribution of *p*-values greater than 0.01, as displayed in Fig 6; for distribution 2 this happens only in the last two groups). The intervals *L*_*i*_ range from *L*_1_ = [115, 5291] to *L*_6_ = [25739, 34378] and to *L*_13_ = [89476, 103767].

**Fig 6:**
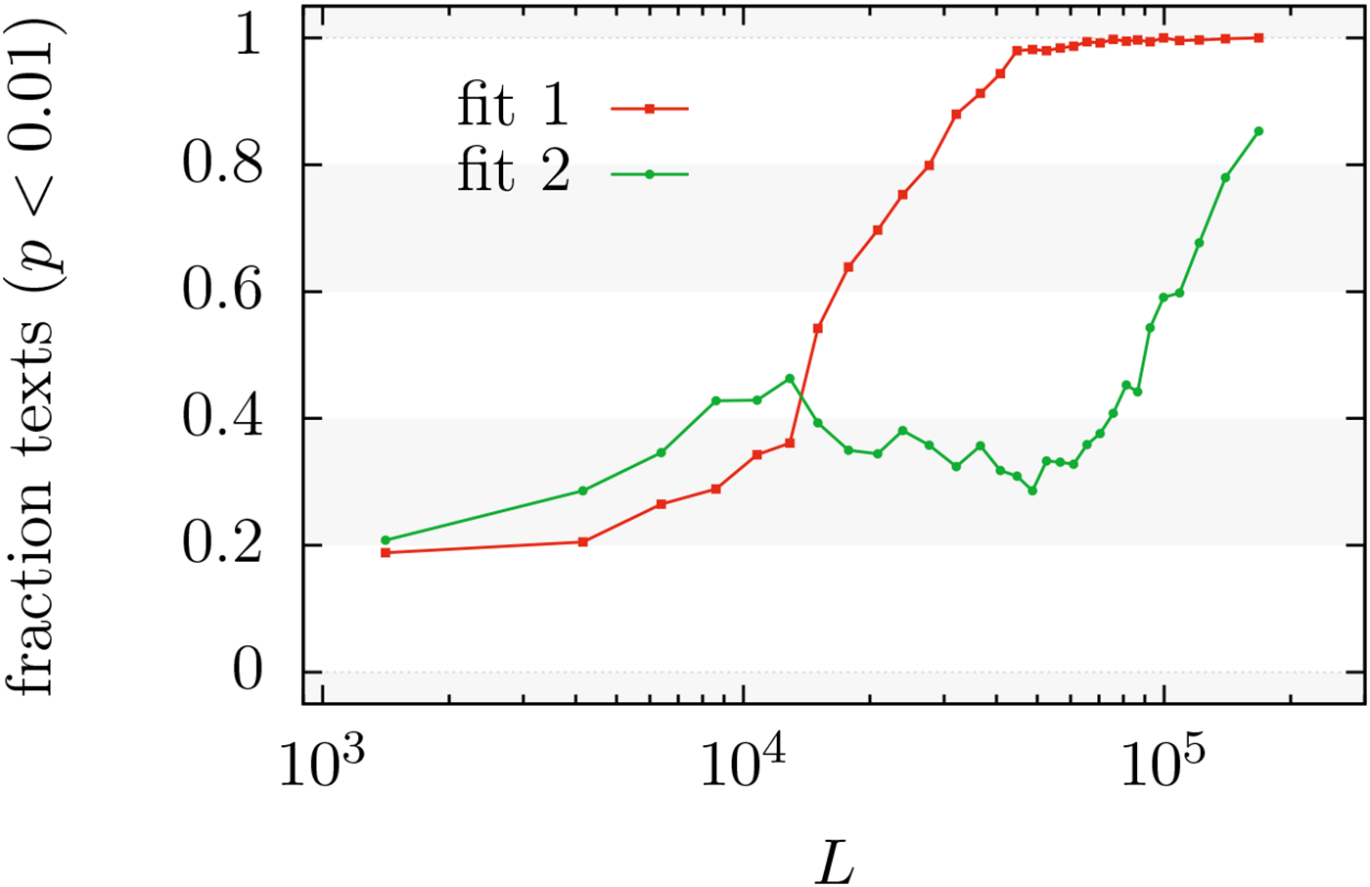
Number of texts with *p*-value near zero (*p* < 0.01) in different ranges of *L* divided by the number of texts in the same ranges, for the fits of distributions *f*_1_ and *f*_2_. Values of *L* denote the geometric mean of ranges containing 1000 texts each. The higher value for the fit of *f*_1_ (except for *L* below about 13000 tokens) denotes its worst performance.

The next question we address is the dependence of the performance of fits on text length *L*. In order to asses this, note that from the shape of the histograms in Fig 2 we can distinguish two groups of texts: those that lie in the zero bin (whose *p*-value is strictly less than 0.01), and the rest. Taking the last group, i.e., texts with *p* ≥ 0.01, and partitioning it into different subsets according to text length (i.e., looking at the distribution of *p* conditioned to *p* ≥ 0.01 for different ranges of *L*), it holds that the shape of the resulting distribution of *p* does not strongly depend on *L*, as shown in Fig 5. In contrast, the number of texts that yield *p*-value near zero certainly varies with *L*, see Fig 6. Therefore, in order to compare the performances of *f*_1_ and *f*_2_ as a function of the text length *L*, it is enough to consider a single value of the significance level (greater than zero) as the results for any other significance level will be the same, in relative terms.

**Fig 7:**
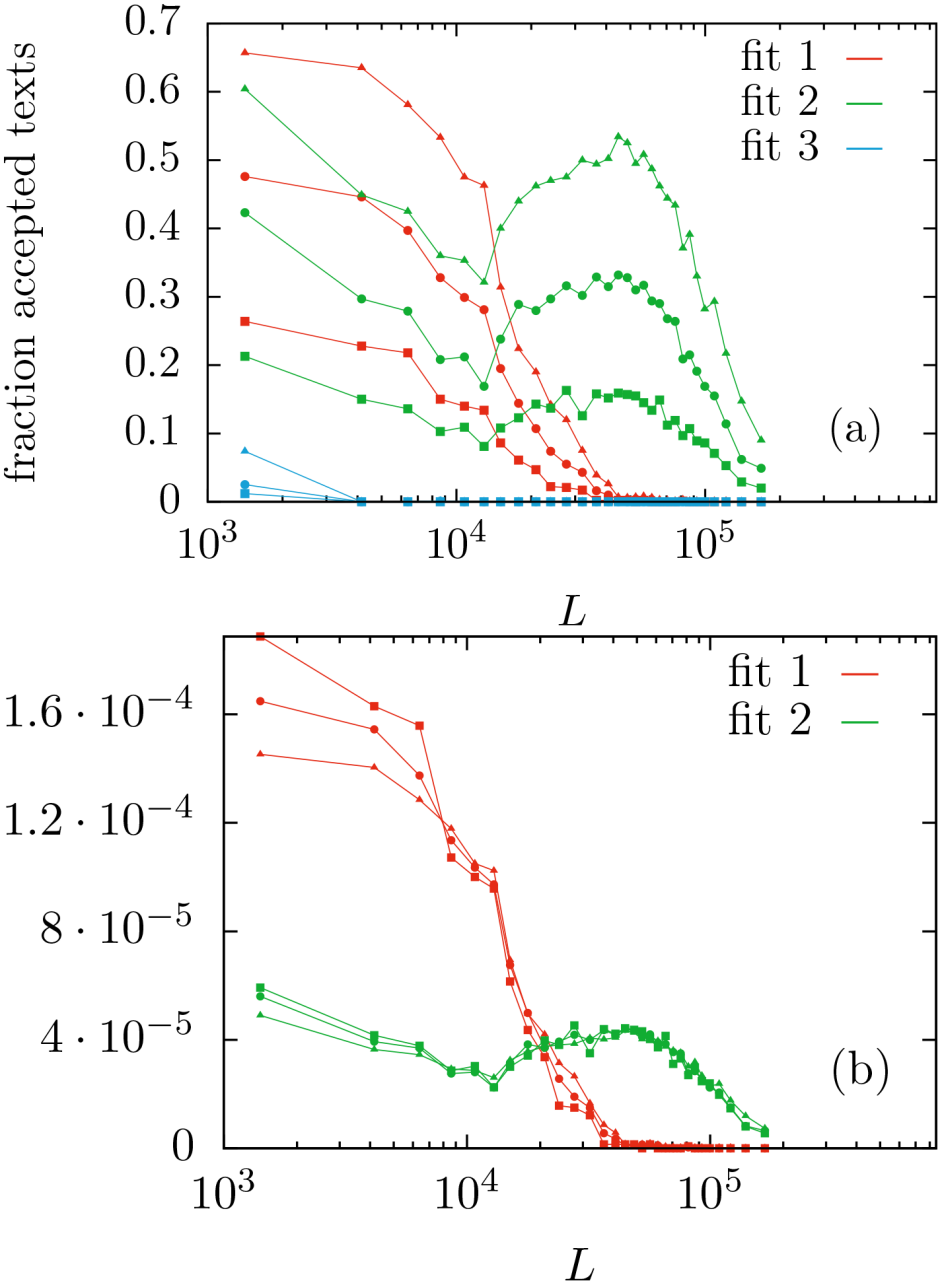
(a) Histograms showing the fraction of accepted texts by the three distributions as a function of their text length, for three different significance levels *p*_0_: 0.05 (upper curves), 0.20 (middle), 0.50 (lower). To be concrete, for each range of *L*, the ratio between the number of texts with *p* ≥ *p*_0_ and the number of texts in that range is calculated. (b) Same curves (removing those for distribution 3) under rescaling. We rescale the *y*—axis by the number of *p* ≥ *p*_0_, in each case, showing that the relative performance of each fit with regard *L* is independent on the significance level. Bins are selected to contain 1000 texts each.

Indeed, Fig 7(a) shows how distribution *f*_1_ fits some more texts than distribution *f*_2_ for small values of *L*, up to about 13000 tokens. But for larger texts, distribution *f*_2_ clearly outperforms distribution *f*_1_, which becomes irrelevant for *L* beyond 100000 (at 0.05 significance level), whereas distribution *f*_2_ is able to fit many texts with *L* larger than 200000. The figure shows that this is the case no matter if the significance level is 0.05, 0.20, or 0.50; the collapse of the curves in Fig 7(b) confirms this fact. From Fig 6 one could infer the same for significance level equal to 0.01. This stability of the performance of the fits for different significance levels arises from the observed fact that the distributions of *p*-values (conditioned to *p* ≥ 0.01) are nearly identical for different *L*, as shown in Fig 5.

Next, we apply the LR test to all texts that have been fitted, considering 0.05 as significance level, by at least one of the two distributions *f*_1_ and *f*_2_. Table 1 merges the results of this test and our previous procedure (based on ML estimation plus the KS test). The total number of texts previously fitted by *f*_1_ or/and *f*_2_ is displayed depending on the sign of the corresponding log-ratio *R*_1,2_. However we must take into account that the sign of the obtained value of *R*_1,2_ could be a product of just statistical fluctuations if the true value were zero and thus, the sign of *R*_1,2_ cannot be trusted in order to discriminate between two models. The probability under the null hypothesis, of obtaining an absolute value of the log-ratio greater than the empirical value |*R*_1,2_| is computed through:

$$p_{LR}\;=\;\text{erfc}\left(\frac{|R_{1,2}|}{\sqrt{2N\sigma^{2}}}\right)$$

where erfc is the complementary error function [45]. We will take as statistically significant those *R*_1,2_ that yield *p*_*LR*_ < 0.05. Equivalently, at 0.05 significance level, *R*_1,2_ is significant if its absolute value is greater than $R_{c}=1.96\sqrt{N\sigma^{2}}$. The results are shown in Table 2

**Table 1:** Number of texts fitted by *f*_1_, *f*_2_ or both.

	*R*_1,2_ > 0	*R*_1,2_ < 0	Total ML-KS
*f*_1_ (exclusively)	3614	81	3695
*f*_2_ (exclusively)	120	11366	11486
*f*_1_ and *f*_2_	431	398	829
Total LR	4165	11845	16010

The number of texts that are fitted by *f*_1_ or *f*_2_ or both at 0.05 significance level of the ML-KS procedure, separated into two columns according to the sign of *R*_1,2_. Positive *R*_1,2_ means that the likelihood for *f*_1_ is greater than that for *f*_2_, and conversely for negative *R*_1,2_. Nevertheless, the sign of *R*_1,2_ is not an indication of significance, for significant LR tests see Table 2.

**Table 2:** Number of texts with a significant LR test.

	*R*_1,2_ > *R*_*c*_	*R*_1,2_ < −*R*_*c*_
*f*_1_ (exclusively)	1666	0
*f*_2_ (exclusively)	0	9423
*f*_1_ and *f*_2_	0	3
Total LR test	1666	9426
None (neither *f*_1_ nor *f*_2_)	510	11431

Number of texts with a significant LR test, at the 0.05 level, either favouring distribution *f*_1_ (*R*_1,2_ > *R*_*c*_) or distribution *f*_2_ (*R*_1,2_ < −*R*_*c*_), for different outcomes of the ML-KS procedure (at the 0.05 level also). Note that these cases correspond to a subset of the previous table. An additional row shows the number of texts that are fitted neither by distribution *f*_1_ nor *f*_2_; notice that in this case a significant LR test does not guarantee a good fit.

Note that the LR test cannot conclude if a fit is good or bad, as it only compares the relative performance of two fits; in other words, if the LR test selects a particular distribution, that distribution can still yield a bad fit, in absolute terms. Anyway, there is no mismatch between the results of both tests: any time the ML-KS method selects one distribution over the other, the LR test either supports the selection or does not give significant results, but it never selects the other option (as shown in Table 2).

**Fig 8:**
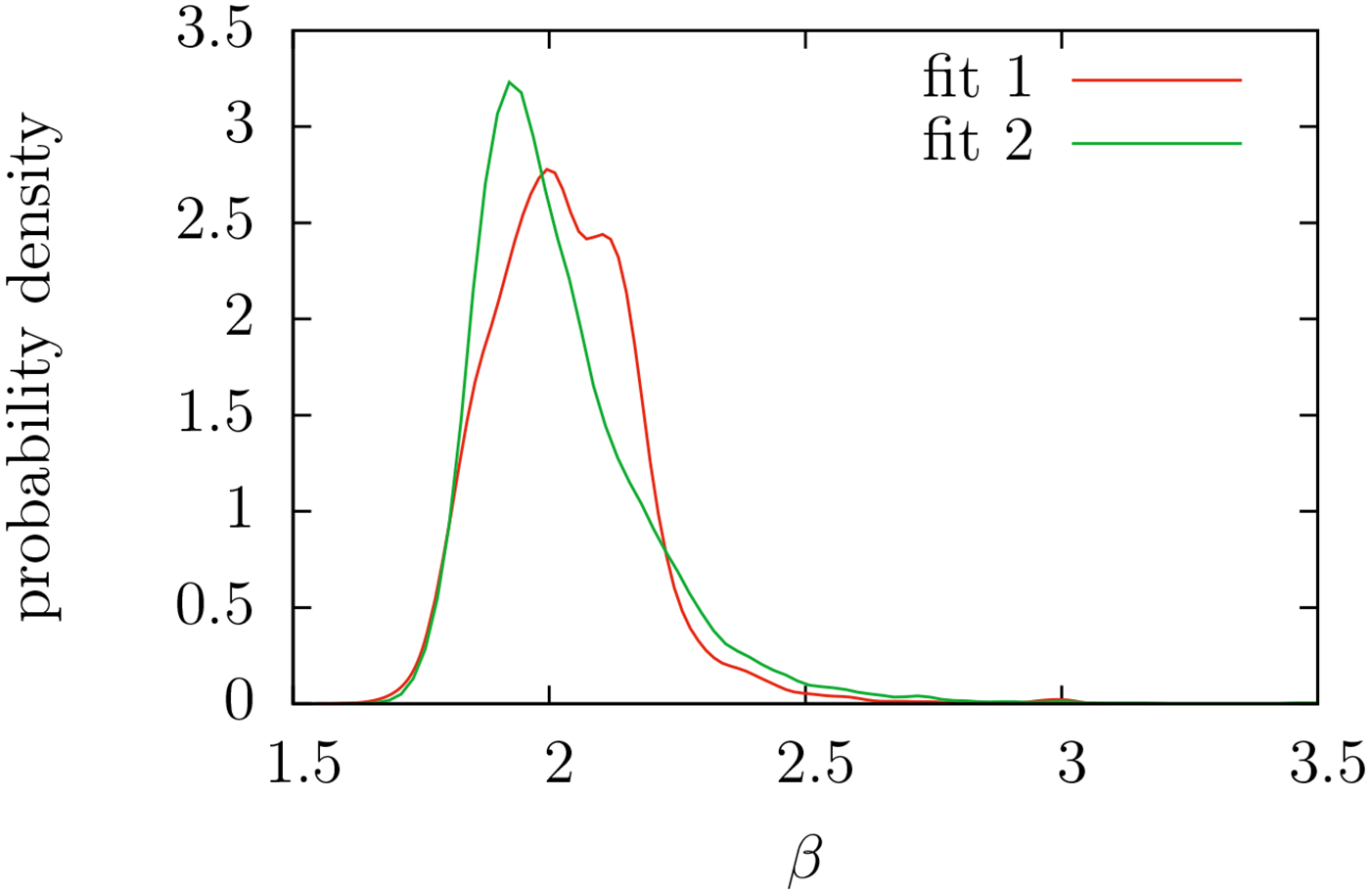
Estimation of the probability density of the Zipf’s exponent *β* for texts yielding *p* ≥ 0.05 in the fits of *f*_1_ and *f*_2_. Curves have been calculated from the histograms via normal kernel smoothing method as implemented in MatLab (*ksdensity* function). Estimated mean and standard deviation of *β* are 2.03 and 0.15 respectively for the fit of *f*_1_, and 2.02 and 0.17 for *f*_2_.

Taking now those texts whose frequency distributions could be approximated by *f*_1_ or *f*_2_, we draw attention to the distribution of the estimated exponents (i.e., the parameter *β*). The original formulation of Zipf’s law implies *β* = 2 and Fig 8 shows that *β* is certainly distributed around 2, with a bell-like shape, and the range of variation is, more or less, between 1.6 and 3. The lower value *β* ≃ 1.6 is in surprising good agreement with the results of the information-theoretic model of Ref. [58], whereas the upper limit *β* ≃ 3 is somewhat larger than the results of that model. Moreover, the distributions of *β* are not symmetric, and the upper limit cannot be defined so sharply as the lower limit. Notice also that texts with limiting values of the exponents do not correspond, in principle, to pathological texts, as claimed in Ref. [58]; rather, for the smallest *β* we find theatre (including Tosltoy and Ibsen) and children books (some by Jane L. Stewart), whereas the largest *β* correspond to short poems.

**Fig 9:**
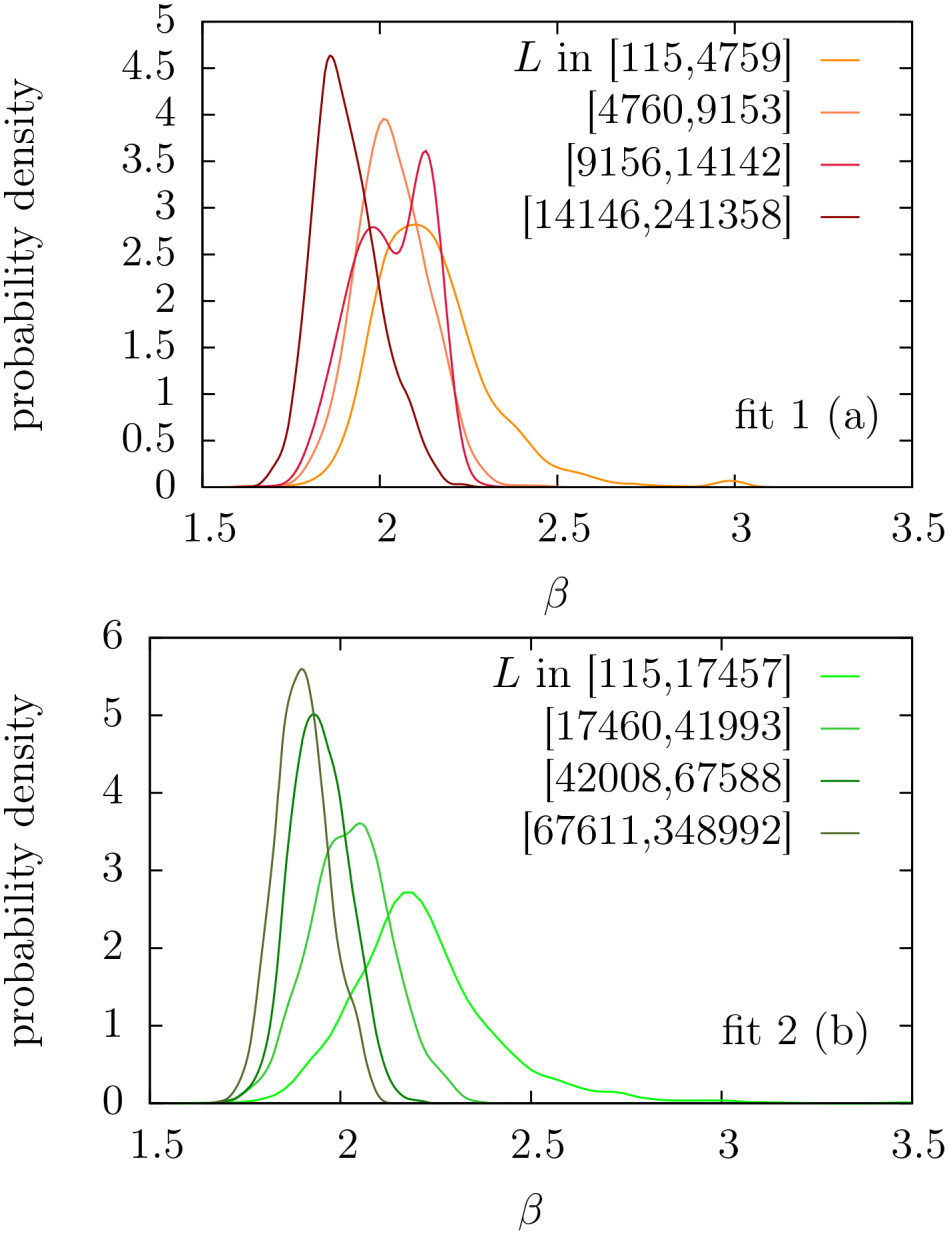
Estimated probability density of *β* for fits with *p* ≥ 0.05, in different length ranges. We have divided both groups of accepted texts into 4 percentiles according to *L*. As in the previous figure, the normal kernel smoothing method is applied. (a) For distribution *f*_1_. (b) For distribution *f*_2_.

**Fig 10:**
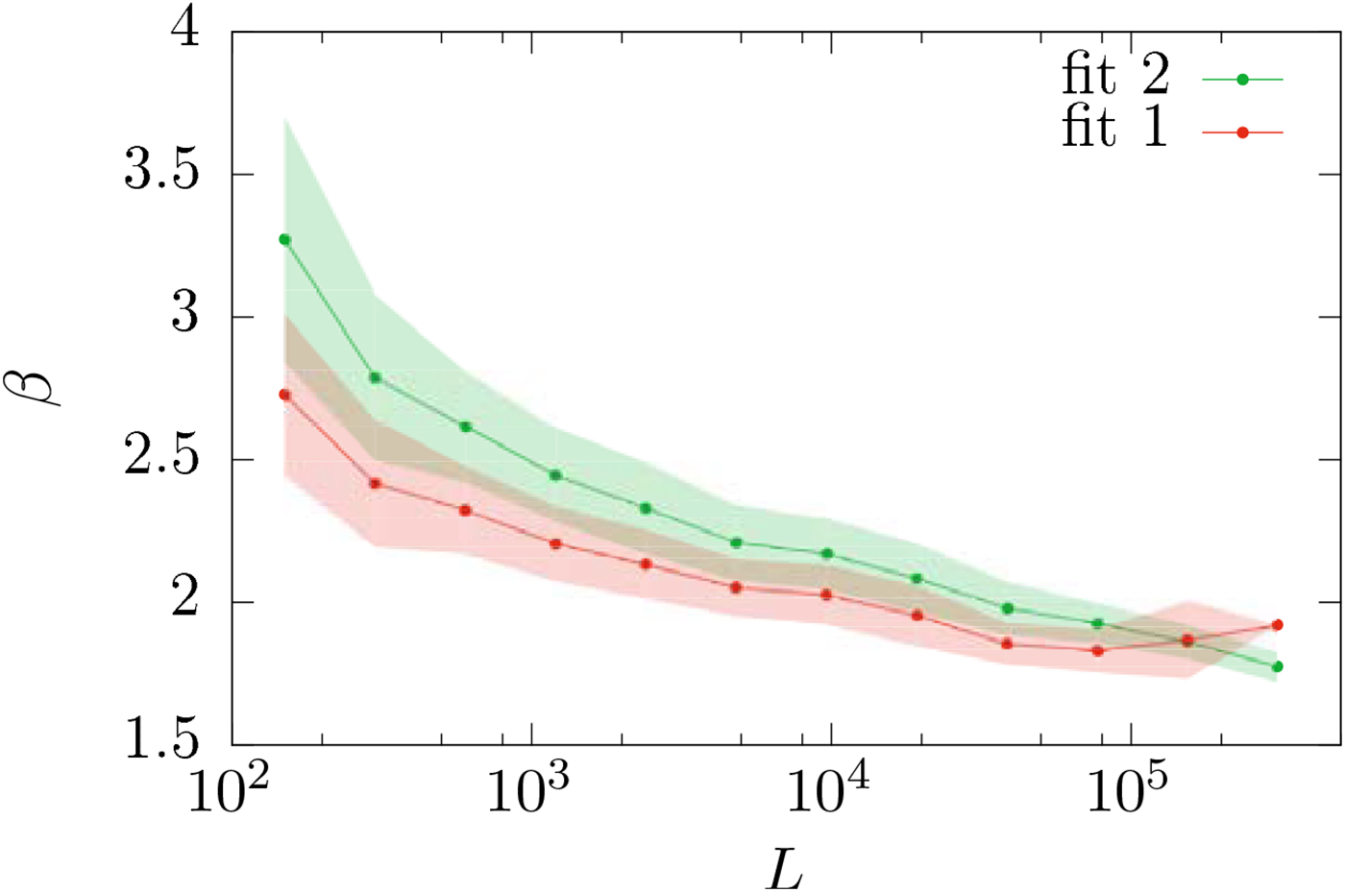
Mean value and standard deviation (represented by the shaded regions) of the distribution of the Zipf’s exponent *β* as a function of text length *L* for distributions *f*_1_ and *f*_2_. Note that the last bin of *f*_1_ contains a single datapoint, and so its standard deviation is not defined.

If we check the effect of the text length *L* in the distribution of *β*, we find a decreasing trend of *β* with *L*, as can be seen in Figs 9 and 10. We have tested that this observation is not an artifact of the fitting method, as synthetic texts generated with fixed *β* do not show this behavior. This trend is not in disagreement with the claims of Ref. [35], where the stability of the exponent *β* was demonstrated for a single growing text (i.e., comparing small parts of a text with the whole). A possible explanation for the decrease of *β* with *L* could be a systematic dependence of *β* with genre, and a bias of *L* with genre. At present we do not have an automatic way to assign genres to texts and we cannot test this hypothesis.

## Discussion and Conclusions

Zipf’s law is probably the most intriguing and at the same time well-studied experimental law of quantitative linguistics, and extremely popular in its wider sense in the science of complex systems. Although the previous literature is vast, as far as we know our work constitutes the first large-scale analysis of Zipf’s law in single (non-aggregated) texts. Thus, we are in a position to make a well-grounded statement about the validity of Zipf’s law in such texts (when those are written in English).

Let us first briefly summarize, however, some key technical points of our study. First, we have analyzed a total of 31 075 English texts from the Project Gutenberg database using rigorous fitting procedures, and have tested how well they are described by three Zipf-like distributions. Our choice of distributions has not been exhaustive; rather, we have limited ourselves to different interpretations of what can be understood as “Zipf’s law”, in the sense of having a perfect power law either in the probability mass function of word frequencies, or in the complementary cumulative distribution function (whose empirical estimation leads to the rank-frequency relation of the sample), or in the rank-frequency relation of an underlying population. Remarkably, the resulting distributions have a unique parameter, *β*, which in all cases is the exponent of an asymptotic power law in the probability mass function of the frequency. It is left to explore how other, more complicated extensions of Zipf’s law perform on this large corpus, but it is obvious that, by including additional parameters, one might provide good fits to a larger number of texts (although in this case, proper model selection will require to balance number of parameters and parsimony).

Our aim in this paper has not been to fit as many texts as possible, but to test the performance of the simplest Zipf-like distributions within a very strict, conservative framework. Indeed, by requiring the three versions of Zipf’s law to hold on the full range of frequencies *n* = 1, 2, … (and not only on the tail of the distribution) we put ourselves in the strictest range of demands. It is hence remarkable that, e.g., at the standard significance level of 0.05, and for text lengths between 10^4^ and 10^5^ word tokens, more than 40% of the considered texts are statistically compatible with the pure power law in the complementary cumulative distribution function represented by distribution *f*_2_ (see Fig 7). So, we can state that, for the corpus under consideration, the most appropriate version of Zipf’s law is given by a probability mass function

$$f\left(n\right)\;=\;\text{Prob}\left[\text{frequency}=n\right]=\frac{1}{n^{\beta -1}}-\frac{1}{{\left(n+1\right)}^{\beta -1}},$$

or, equivalently, by a complementary cumulative distribution function

$$S\left(n\right)\;=\;\text{Prob}\left[\text{frequency}\geq n\right]=\frac{1}{n^{\beta -1}}.$$

Due to the broad coverage of the Project Gutenberg corpus we speculate that this distribution should fit a large fraction of generic (non-technical) English texts. Of course, testing this speculation in front of all possible corpora is an impossible task.

We have also shown that our conclusions regarding the *relative* performance of a pure power law in the probability mass function, given by distribution *f*_1_, versus distribution *f*_2_ are robust with respect to changes in the significance level: about twice as many texts are statistically compatible with distribution *f*_2_ than those compatible with *f*_1_, at any significance level (obviously, in absolute terms, the number of accepted texts varies with the significance level). Hence we can conclude that distribution *f*_2_ gives a better description of English texts than distribution *f*_1_, at least for the corpus considered in this work.

We may speculate that the predominance of *f*_2_ in front of *f*_1_ (and in front of *f*_3_) may be a peculiarity of the English language, caused by its poor inflectional morphology. Indeed, the difference between these distributions is in the lowest frequencies (mainly *n* = 1, *n* = 2…). Languages with a richer inflectional morphology should yield a larger proportion of low-frequency words (in comparison to the other words) than English, favoring perhaps *f*_1_ in front of *f*_2_. This analysis is left for future studies. Lemmatization of texts [6] would be very helpful to test this speculation, but at present we cannot afford large-scale lemmatization.

Another conclusion is that distribution *f*_3_, first derived by Mandelbrot [40], is irrelevant for the description of texts in this corpus. Finally, we have corroborated that the exponent *β* of Zipf’s law certainly varies from text to text, as had been previously claimed using other approaches for defining what Zipf’s law is [5, 6]. Interestingly, the value *β* = 2 originally proposed by Zipf himself is among the most frequent ones.

We believe that our analysis constitutes a major advancement in the understanding of Zipf’s law. It is astonishing how good the simplest one-parameter Zipf-like distributions perform on such a large set of texts, particularly with the strict set of requirements we have imposed. This is in sharp contrast for instance with Zipf’s law in demography [59] and in the distribution of income [60], where the power law seems to be valid only for the tail corresponding to the largest sizes, as it happens also for the distribution of word frequency in large text corpora, as mentioned above [2, 36–39].

Zipf’s law has been subject to much debate, and will probably continue to be so for many years. Indeed, one can always cast doubt on its validity on the basis of some particular examples. Yet it seems clear to us that, in our modern times of big data and large computational capabilities, more efforts should be put towards large-scale analysis of Zipf’s law. We hope this paper constitutes a first step in this direction.

## Appendix: Simulation of Discrete Zipf-like Distributions

As part of the testing procedure, we need simulated samples from *f*_1_, *f*_2_, and *f*_3_, which are discrete distributions defined for *n* = *a*, *a* + 1, …. We will give the recipe of simulation for an arbitrary positive integer value of the lower cut-off *a*. It is simpler to start with *f*_2_, as this is used as an auxiliary distribution in the simulation of the other two.

### Simulation of *f*_2_

Fixed *a* and given the parameter *β*, we want a set of random numbers whose complementary cumulative distribution function is a discrete power law: *S*_2_(*n*) = (*a*/*n*)^*β*−1^. For that, we first generate a random number *u* from a uniform distribution in the interval (0, *u*_*max*_), with *u*_*max*_ = 1/*a*^*β*−1^. The inversion method [56, 57] guarantees that if we take *x* = 1/*u*^1/(*β*−1)^, the values of *x* yield a continuous power law with $S_{2}^{c}\left(x\right)={\left(a/x\right)}^{\beta -1}$, for *x* ≥ *a*, where the superscript *c* distinguishes the continuous distribution from its discrete analogous one. So, taking *n* equal to the integer part of *x*, i.e., *n* = int(*x*), yields a discrete distribution with *S*_2_(*n*) = (*a*/*n*)^*β*−1^, as desired. This is so because, for any *X*, int(*X*) ≥ *n* is equivalent to *X* ≥ *n* for *n* integer. In a recipe:

1. generate *u* from a uniform distribution in (0, 1/*a*^*β*−1^],
2. calculate *x* = 1/*u*^1/(*β*−1)^,
3. take *n* = int(*x*).

By means of the so-called rejection method [56, 57], simulated integers distributed following *f*_2_ can be used for the simulation of integers following *f*_1_ or *f*_3_. The key point to achieve a high performance in the rejection method is to use a “good” auxiliary function, i.e., one that leads to a low rejection rate. This is certainly the case in our framework, as explained below.

### Simulation of *f*_1_

In this case, the steps are:

1. generate *n* from *f*_2_(*n*),
2. generate *v* from a uniform distribution in the unit interval,
3. *n* is accepted if
  $$v\;\leq \frac{f_{1}\left(n\right)}{f_{2}\left(n\right)C},$$
  and rejected otherwise, where *C* is the rejection constant given by *C* = max_*n* ≥ *a*_{*f*_1_(*n*)/*f*_2_(*n*)}.

Note that the rejection method [56, 57] guarantees that the resulting (non-rejected) *n* will be distributed according to *f*_1_.

It is easy to check that the maximum of *f*_1_/*f*_2_ is reached at *n* = *a* as this is a decreasing function [41]. The acceptance condition above can be simplified by taking *τ* = (1 + *n*^−1^)^*β*−1^, and *b* = (1 + *a*^−1^)^*β*−1^, then, the condition becomes:

bvn(τ-1)≤a(b-1)τ,

which is devoid of the calculation of the Hurwitz-zeta function. This is a generalization for *a* > 1 of the method of Ref. [56]. The choice of *f*_2_ as the auxiliary distribution function is justified by the small value that *C* takes, as this is the expected number of generated values of *n* until we accept one. For instance, for *β* = 2 and *a* = 1 we get *C* = 1.2158.

### Simulation of *f*_3_

Proceeding similarly, we get in this case low values of *C* = max_*n* ≥ *a*_{*f*_3_(*n*)/*f*_2_(*n*)} as well (we get *C* = 2 in the limit *β* → 2 for *a* = 1). The maximum of *f*_3_(*n*)/*f*_2_(*n*) is numerically seen to be reached at *n* = *a*. In summary, the steps are:

1. generate *n* from *f*_2_(*n*)
2. generate *v* from a uniform distribution in the unit interval
3. *n* is accepted if
  $$vf_{2}\left(n\right)\;\leq a\left(1-{\left(\frac{a}{a+1}\right)}^{\beta -1}\right)\frac{\Gamma (n-(\beta -1))}{\Gamma (n+1)}\frac{\Gamma (a)}{\Gamma (1+a-\beta )}$$
  and rejected otherwise.
